# The modulation effect of longitudinal acupuncture on resting state functional connectivity in knee osteoarthritis patients

**DOI:** 10.1186/s12990-015-0071-9

**Published:** 2015-10-29

**Authors:** Xiaoyan Chen, Rosa B. Spaeth, Sonya G. Freeman, Donna Moxley Scarborough, Javeria A. Hashmi, Hsiao-Ying Wey, Natalia Egorova, Mark Vangel, Jianren Mao, Ajay D. Wasan, Robert R. Edwards, Randy L. Gollub, Jian Kong

**Affiliations:** Department of Psychiatry, Massachusetts General Hospital, Charlestown, MA USA; Mass General Orthopaedics Sports Performance Center, Massachusetts General Hospital, Foxborough, MA USA; MGH/MIT/HMS Athinoula A. Martinos Center for Biomedical Imaging, 120, 2nd Ave. Suite 101, Charlestown, MA 02129 USA; Department of Radiology, Massachusetts General Hospital, Charlestown, MA USA; Departments of Anesthesiology, Massachusetts General Hospital, Charlestown, MA USA; Departments of Anesthesiology, Perioperative, and Pain Medicine, and Psychiatry, Brigham and Women’s Hospital and Harvard Medical School, Boston, MA USA; Departments of Anesthesiology and Psychiatry, University of Pittsburgh School of Medicine, Pittsburgh, PA USA

**Keywords:** Acupuncture, Knee osteoarthritis, Chronic pain, Resting state fMRI, Independent component analysis (ICA), Knee injury and Osteoarthritis Outcome Score (KOOS), Right frontoparietal network, Executive control network

## Abstract

Recent advances in brain imaging have contributed to our understanding of the neural activity associated with acupuncture treatment. In this study, we investigated functional connectivity across longitudinal acupuncture treatments in older patients with knee osteoarthritis (OA). Over a period of 4 weeks (six treatments), we collected resting state functional magnetic resonance imaging (fMRI) scans from 30 patients before and after their first, third and sixth treatments. Clinical outcome showed a significantly greater pain subscore on the Knee Injury and Osteoarthritis Outcome Score (KOOS) (indicative of improvement) with verum acupuncture than with sham acupuncture. Independent component analysis (ICA) of the resting state fMRI data showed that the right frontoparietal network (rFPN) and the executive control network (ECN) showed enhanced functional connectivity (FC) with the rostral anterior cingulate cortex/medial prefrontal cortex, a key region in the descending pain modulatory system, in the verum groups as compared to the sham group after treatments. We also found that the rFPN connectivity with the left insula is (1) significantly associated with changes in KOOS pain score after treatments, and (2) significantly enhanced after verum acupuncture treatments as compared to sham treatment. Analysis of the acupuncture needle stimulation scan showed that compared with sham treatment, verum acupuncture activated the left operculum/insula, which also overlaps with findings observed in resting state analysis. Our results suggest that acupuncture may achieve its therapeutic effect on knee OA pain by modulating functional connectivity between the rFPN, ECN and the descending pain modulatory pathway.

Clinical trial number: NCT01079390

**Electronic supplementary material:**

The online version of this article (doi:10.1186/s12990-015-0071-9) contains supplementary material, which is available to authorized users.

## Background

Osteoarthritis (OA) is a major public health problem among the elderly and is associated with considerable disability [1]. The knee is one of the most common body locations for OA. A recent analysis of data from the National Health and Nutrition Examination Survey III indicated that about 35 % of women and men aged 60 years and above had radiographic knee OA [2].

OA is a complex chronic pain condition due in part to both nociceptive and neuropathic mechanisms [3]. Previous studies have demonstrated central sensitization in OA patients [4–6] and increased activity in the periaqueductal grey (PAG) is associated with stimulation of the skin in referred pain areas of patients [6], indicating a pathology change in the central nervous system of OA patients.

Despite the high prevalence rate of OA, the treatment of OA is far from satisfactory [7, 8]. Pharmacological treatment of knee OA is often ineffective with unwanted and dangerous side effects [8, 9].

Arguably, acupuncture may be a promising treatment option for knee OA due to the effectiveness of the pain relief it provides [8, 10–14], and the rarity of adverse effects [15–18]. Nevertheless, the mechanisms underlying the effects of acupuncture treatment in knee OA patients are still poorly understood. As a unique treatment modality, studies have shown that acupuncture may produce an analgesic effect through the endogenous descending pain modulatory system [19–22].

Brain imaging studies have shown that acupuncture needle stimulation [23–27] can evoke widespread brain activity changes, and modulate the functional connectivity (FC) of the pain processing network [28–35], which opens a new window for understanding the central mechanism of acupuncture treatment. In addition, investigators have found abnormal or disrupted FC in patients with various chronic pain disorders [36–41], and that FC can be modulated by analgesics in both healthy [42] and chronic pain patients [43]. These studies suggested that FC may be a useful tool in both acupuncture and chronic pain research [34].

In the present study, we investigated changes in FC in knee OA patients across longitudinal acupuncture treatments. One challenge of acupuncture research is variability in the prescription of acupoints for the same disorders, i.e., the number of acupuncture acupoints (needles) used during treatment. As acupuncture is an invasive treatment, fewer needles are always preferred in acupuncture practice. In this study, in addition to comparing the effect/mechanism of real and sham acupuncture treatment, we also attempted to explore the effects of the number of acupoints after controlling for the total stimulation time by dividing the verum acupuncture group into two subgroups: low dose (two local acupoints around the knee), and high dose (two local acupoints around the knee plus an additional four local and distant acupoints).

Knee OA patients were randomized to receive either high dose verum acupuncture, low dose acupuncture or sham acupuncture. Using a longitudinal treatment design [8], each patient received 6 acupuncture treatments over 4 weeks. We measured brain activity during acupuncture needle stimulation and resting state functional connectivity using functional magnetic resonance imaging (fMRI) during the first, third and sixth treatment (Additional file 1: Figure S1a). We also administered the Knee Injury and Osteoarthritis Outcome Score (KOOS) [44] before and after the six-session acupuncture treatment period.

## Results

Forty-four acupuncture naïve patients (19 females) aged 43–70 with a diagnosis of chronic osteoarthritis in the right and/or left knee participated in the study. Of the 44 patients who enrolled, 30 (13 females) completed all study procedures. Fourteen subjects did not complete the study due to problems with scheduling (6), ineligibility at screening (3), disinterest (2), claustrophobia (1), or inability to adhere to study requirements in the scanner (2). Of the 14 subjects who dropped out of the study, four dropped out after randomization (2 from the low dose verum acupuncture group and 2 from the high dose verum acupuncture group).

### Sensation evoked by acupuncture treatment, clinical outcomes and blindness

As a treatment quality control, we measured the deqi sensation evoked by acupuncture treatment using the MASS [45, 46]. The average total MASS score (sum of the intensities of each sensation) differed significantly across the acupuncture treatment groups [high (12.95 ± 7.55) vs. low (15.22 ± 11.90) vs. sham (4.82 ± 3.79)] [*F*(2,27) = 4.21, p = 0.026]. Post hoc analysis showed the following differences: high vs low p = 0.55, high vs sham p = 0.04; low vs sham p = 0.01. There was no significant difference between the high and low dose acupuncture groups. A previous study by our group reported more details regarding the deqi sensations evoked by different treatment modalities in this sample [45].

We measured clinical outcomes using the KOOS [44], which is comprised of 5 subscales: (1) pain, (2) other symptoms, (3) function in daily living, (4) function in sport and recreation, and (5) knee-related quality of life at the beginning and end of treatment (Table 1). Because this study focused on brain networks involved in the pain process, we only reported the comparisons of KOOS pain changes before and after treatments in different groups. Please see our previously published paper on the comparison of other outcomes [45]. We found that both high and low dose verum acupuncture groups showed significant improvement or a trend (post- minus pre-treatment) as compared with sham acupuncture [p = 0.07 (high dose vs sham), p = 0.04 (low dose vs sham)]. The improvement in the two real acupuncture treatment groups was similar (p = 0.90, low dose vs high dose). Pooling the data from two real acupuncture groups using repeated measurements showed that there was a significant interaction between acupuncture mode (real vs. sham) and time (baseline vs. endpoint) for pain [F(1,28) = 5.596, p = 0.025]. Since our primary outcome, the pain KOOS subscale, showed the most significant difference between verum and sham acupuncture groups, and KOOS pain is the most relevant score for central pain processing, we used the KOOS pain subscale for the following fMRI regression analysis.

**Table 1:** Demographics and characteristics at baseline, and clinical outcomes before and after longitudinal acupuncture treatments

Mean ± SD	All	High dose	Low dose	Sham
N (F)	30 (13)	10 (2)	10 (7)	10 (4)
Age	58 ± 8	60 ± 9	58 ± 8	54 ± 7
Duration (treated knee, years)*	11 ± 8	10 ± 7	6 ± 6	16 ± 8
Pain				
Baseline	56 ± 14	59 ± 13	53 ± 9	56 ± 19
Post-treatment	64 ± 14	70 ± 16	66 ± 11	56 ± 12
Symptoms				
Baseline	53 ± 16	57 ± 19	48 ± 11	54 ± 18
Post-treatment	58 ± 15	60 ± 20	58 ± 15	55 ± 11
Adjusted daily living				
Baseline	64 ± 15	66 ± 12	61 ± 14	64 ± 20
Post-treatment	72 ± 16	74 ± 18	76 ± 12	65 ± 15
Function in sport				
Baseline	30 ± 23	30 ± 18	31 ± 19^†^	29 ± 31
Post-treatment	41 ± 26	49 ± 33	48 ± 18	28 ± 21
Quality of life				
Baseline	39 ± 15	42 ± 17	38 ± 14	36 ± 16
Post-treatment	38 ± 18	44 ± 20	41 ± 16	29 ± 17

Results reported as mean ± SD

Please note that greater score on the KOOS indicates improvement

* Significant main effect of group (high vs. low vs. sham)

^†^N = 9 (one subject missing KOOS sport subscale score)

At the end of the study, we also assessed how well patients’ blinding was maintained throughout the study. Ninety percent (n = 27) of the subjects believed that the needle was inserted into the skin in every session. The 3 subjects who believed that the needle was not inserted were in the real acupuncture (low dose) group.

### Independent component analysis

ICA identified three resting state networks (i.e., the rFPN, ECN and somato-sensory network (SMN) as shown in Additional file 2: Figure S2). SMN includes the supplementary motor area, sensorimotor cortex, and secondary somatosensory cortex and insula. The ECN covers several lateral and medial-frontal areas, including the anterior cingulate and paracingulate. The rFPN covers several frontoparietal areas including the bilateral dorsal lateral prefrontal gyri and the inferior and superior parietal lobules [47]. These networks are consistent with findings from previous published results [48].

### Functional connectivity: comparing verum to sham group

**Fig. 1:**
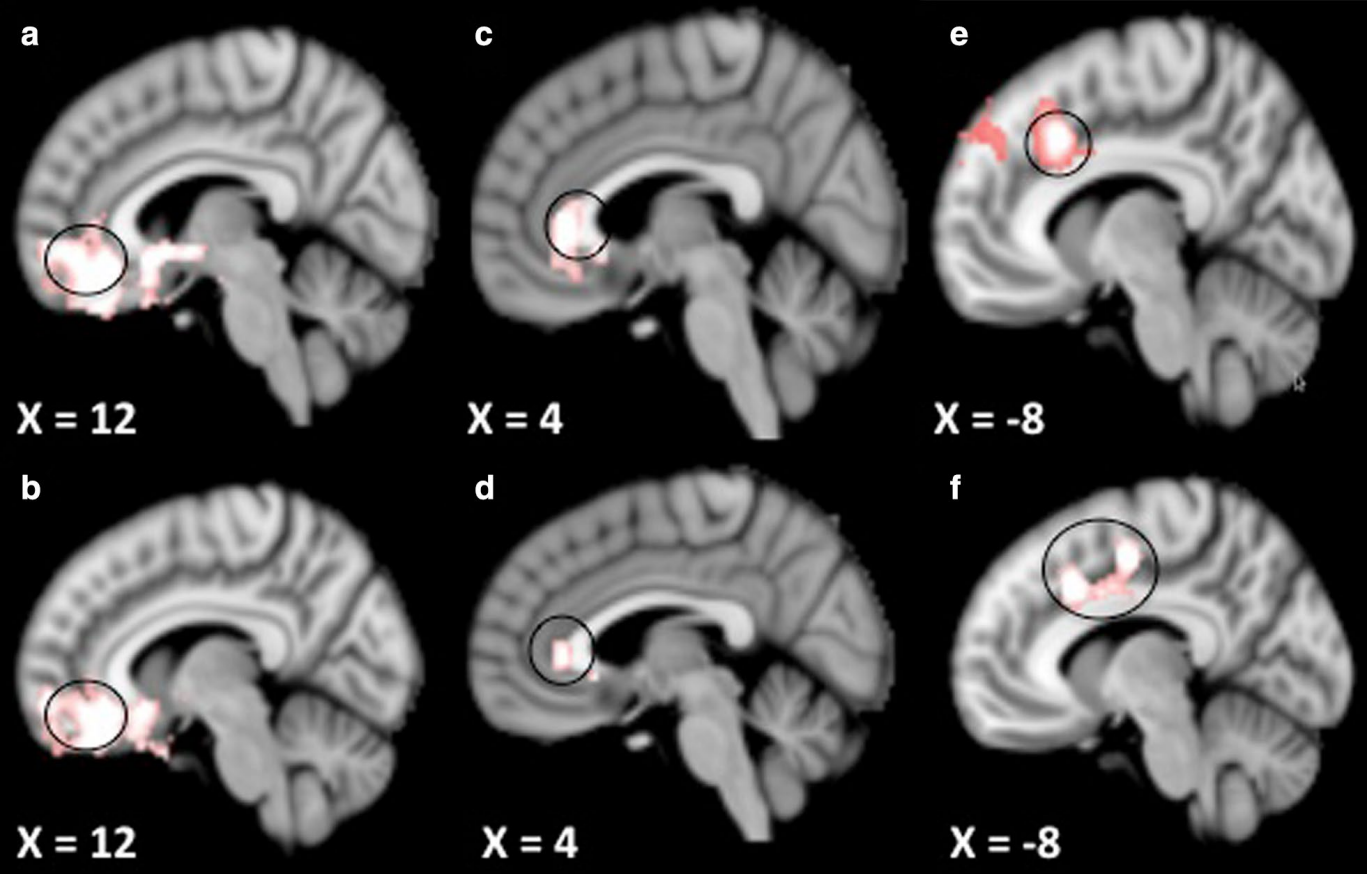
Comparison of the pre-acupuncture scans in treatments 6 and 1. **a** Connectivity between the right frontoparietal network and the rACC increases more in the verum group than in the sham group. (X = 12) **b** Results in **a** controlled for age and duration of pain. **c** Executive control network showed stronger connectivity with rACC after treatment in the verum group than in the sham group. (X = 4) **d** Results in **c** controlled for age and duration of pain. **e** Sensory-motor network showed reduced connectivity with dACC after real acupuncture treatment compared with sham group. (X = −8) **f** Results in **e** controlled for age and duration of pain

The comparison in FC changes from pre-acupuncture treatment 1 to pre-acupuncture treatment 6 between the verum and sham groups showed stronger rFPN connectivity with the bilateral rostral anterior cingulate gyrus (rACC)/medial prefrontal cortex (MPFC), parahippocampus, right insula, superior and inferior temporal gyrus, inferior frontal gyrus, left insula/putamen and thalamus in the verum group than in the sham group (Table 2). Figure 1a identifies the rACC/MPFC as a representative brain region that showed significant difference between the verum and sham acupuncture groups. Further analysis showed that Z connectivity values (3 mm sphere around the peak) of the rACC increased gradually across treatment sessions in the verum group but not the sham group. Repeated measures ANOVA showed that there was a highly significant interaction between acupuncture mode (verum vs. sham) and time (across all three treatment sessions) for rACC/MPFC Fisher Z values [F(2,27) = 13.403, p < 0.0001]. After including age and duration of pain as covariates of no interest, the main finding that the verum group showed stronger rFPN connectivity with rACC/MPFC than did the sham group comparing FC changes from treatment 1 to treatment 6 remained valid (Fig. 1b).

**Table 2:** Regions that show connectivity change with pre-acupuncture resting state network after longitudinal acupuncture treatment (pre-treatment 6—pre-treatment 1) comparing acupuncture to sham group

Network	Group	Area	Z score	Peak coordinate (x, y, z)
Right frontoparietal network	Acu > Sham	rACC/MPFC	4.45	12, 36, −12
		Right parahippocampus	3.58	32, 0, −18
		Right midinsula/precentralgyrus	3.98	52, −2, 2
		Right sup temporal/right inf temporal gyrus	4.15	38, 4, −34
		Right inferior frontal/mid frontal gyrus	3.25	50, 32, −4
		Left midinsula	4	−24, 4, 6
		Left thalamus	3.77	−22, −18, 10
		Left claustrum/parahippocampus	3.4	−38, −12, −14
		Left sup temporal gyrus	3.46	−50, −6, 0
	Sham > Acu	None		
Executive control network	Acu > Sham	rACC	3.95	4, 32, 2
		Left MPFC	3.4	−16, 56, −8
		Left insula	3.72	−46, 2, 2
		Left inferior frontal gyrus	3.33	−42, 36, 6
	Sham > Acu	None		
Somatosensory network	Acu > Sham	None		
	Sham > Acu	Dorsal ACC	4.74	−8, 18, 38
		Bilateral sup frontal/left middle frontal/medial frontal gyrus	3.6	−20, 48, 42

The ECN showed stronger connectivity with rACC/MPFC after verum acupuncture compared to sham acupuncture (Fig. 1c). Further analysis found that in the verum group, but not in the sham group, Z connectivity values of the rACC/MPFC increased gradually across treatment sessions. Repeated measures of ANOVA on Z values (3 mm sphere around the peak) showed that there was a highly significant interaction between acupuncture mode (verum vs. sham) and time (across all three treatment sessions) for the rACC Z value [F(2,27) = 11.85, p < 0.0001]. Other regions that showed significantly greater connectivity with the ECN in the verum group included the left mid insula and inferior frontal gyrus (Table 2). After controlling for age and duration of pain, the results remained the same—stronger connectivity between the ECN and the rACC/MPFC after acupuncture treatments was observed in the verum group relative to the sham group (Fig. 1d).

The SMN showed reduced connectivity with the dorsal ACC (dACC) in the verum group as compared to the sham group (Fig. 1e). Extracted Z values (3 mm sphere around the peak) from the regions showed a gradual decrease in pre-treatment resting state connectivity values across treatment sessions for the verum group, but not for the sham group. Repeated measures ANOVA showed that there was a highly significant interaction between acupuncture mode (verum vs. sham) and time (across all three treatment sessions) for the dACC Z value [F(2,27) = 12.361, p < 0.0001]. Other regions that showed reduced connectivity with the SMN included the bilateral superior frontal, the left middle frontal, and the medial frontal gyrus (Table 2). Controlling for age and duration of pain did not change the results, namely, reduced connectivity between the SMN and the dACC in the verum group compared to the sham group (Fig. 1f).

### Association between functional connectivity and clinical outcome

**Fig. 2:**
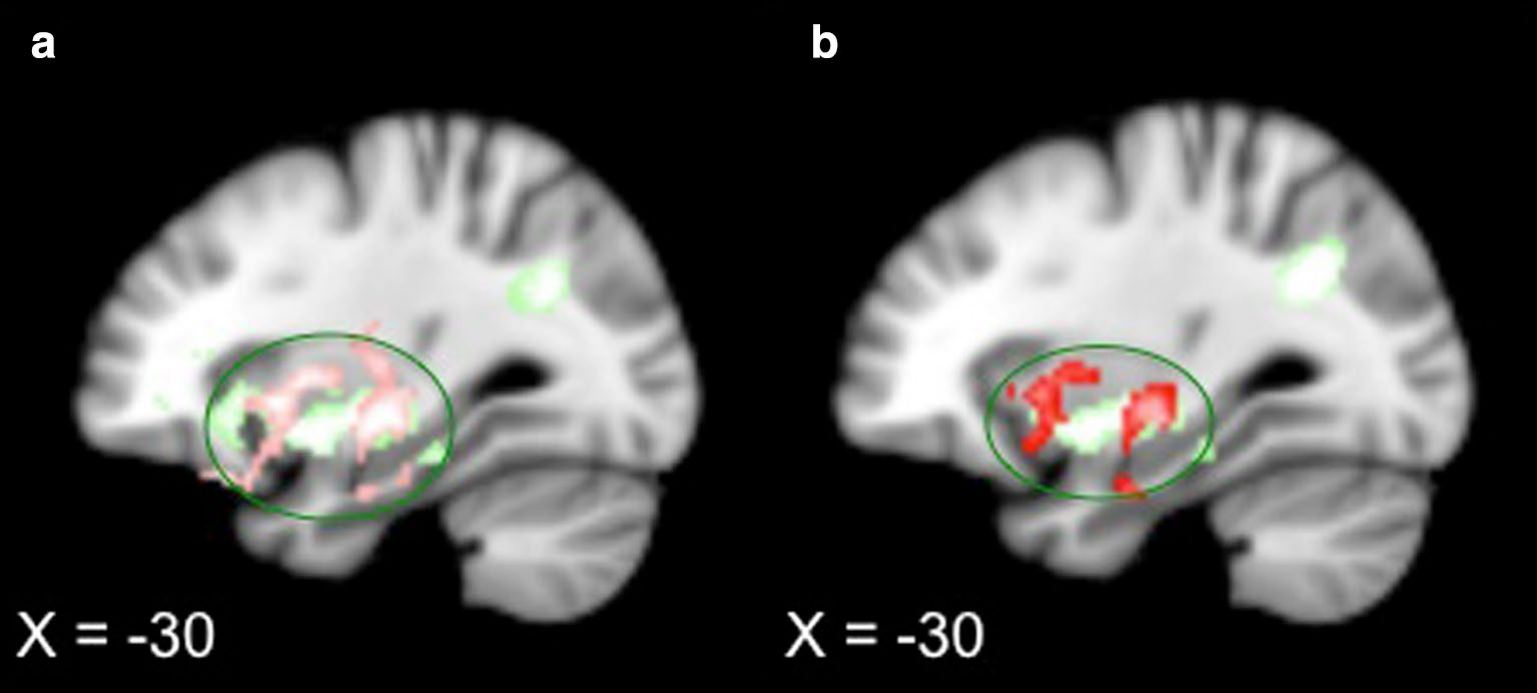
**a** Shown in *green*: after treatment, the increase in functional connectivity between the rFPN and left insula/putamen positively correlated with the change in KOOS pain score. Shown in *red:* the verum group showed significant increase in connectivity between the rFPN and left insula/putamen compared to sham group. (X = −30) **b** Results in **a** after adjusting for age and duration of pain. The comparison between verum and sham shown in *red* were at a less conservative threshold of voxel-wise Z > 1.96 and a corrected cluster significance threshold of P < 0.05

To assess the association between the changes in resting state FC and the corresponding changes in clinical outcome following acupuncture treatment, we applied a regression analysis between the changes of pre-treatment resting state FC maps between treatments 1 and 6 and the corresponding changes in KOOS pain scores (post–pre) for all patients who completed the study. Results showed that the connectivity between the rFPN and the left insula/putamen increased after acupuncture treatment when the change in KOOS pain scores (post–pre) increased (greater KOOS pain score indicates improvement) (r = 0.61, p < 0.001) (Fig. 2a shown in green). Interestingly, this region overlapped with the findings from the group comparison between verum and sham acupuncture, i.e., the FC between the rFPN and left insula significantly increased after treatments in the verum acupuncture group as compared to the sham group (Fig. 2a shown in red). This result further confirmed the important role of the rFPN network in acupuncture modulation.

After adjusting for age and duration of pain, the rFPN and the left insula/putamen connectivity change after acupuncture treatment remained positively correlated with the change in KOOS pain scores (post–pre) (Fig. 2b shown in green). Moreover, the FC between the rFPN and left insula remained increased after treatments in the verum groups as compared to the sham group with a slightly less stringent threshold (voxel-wise cluster forming threshold of Z > 1.96 and a corrected cluster significance threshold of P < 0.05) (Fig. 2b shown in red).

Other regions that showed a treatment-related increase in connectivity with the rFPN coupled with an increase in KOOS pain score include the temporal gyrus, parahippocampus, and cerebellum.

The ECN showed stronger connectivity with the right middle frontal gyrus coupled with an increase in KOOS pain score. The SMN did not show any significant changes in connectivity that correlated with a change in KOOS pain score (Table 3).

**Table 3:** Regions in which treatment-related changes in connectivity (pre-treatment 6–pre-treatment 1) were positively correlated with change in clinical KOOS pain (post–pre)

Clinical outcome	ICA networks	Area	Z value	Peak coordinate
KOOS change in pain	Right frontoparietal network	Left anterior insula	4.06	−34, 30, 8
		Left posterior insula/left posterior parietal operculum	2.9	−52, −32, 20
		Left putamen	3.94	−28, −10, −8
		Left superior temporal/left mid temporal gyrus	4.02	−50, −52, 20
		Left parahippocampus	3.32	−34, −32, −18
		Cerebellum	4.39	18, −48, −38
	Executive control network	Right mid frontal gyrus	3.99	38, 20, 32
	Sensory-motor network	None	None	None

We observed similar results using the same analysis with a lower threshold (voxel-wise cluster forming threshold of Z > 1.96 and a corrected cluster significance threshold of P < 0.05) for 20 patients who only received verum acupuncture treatments.

### Result of task related fMRI data analysis

**Fig. 3:**
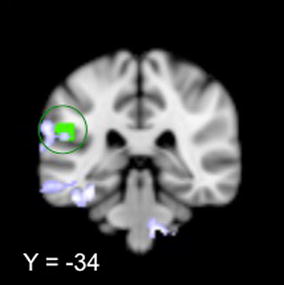
Shown in *green*: during acupuncture needle stimulation, the left posterior parietal operculum showed more activation in the verum group than in the sham group. Shown in *blue*: after treatment, the change (increased) in connectivity between the right frontoparietal network and left posterior parietal operculum correlates with the change in KOOS pain in patients (Y = −34)

Verum acupuncture needle stimulation produced significant fMRI signal increases in the left posterior operculum/secondary somatosensory cortex (S2) (x = 66, y = −24, z = 40, 101 voxels) (Fig. 3 shown in green) and the cuneus (x = 3, y = −96, z = 0, 180 voxel) compared to sham treatment. Interestingly, we found that the increased fMRI signal in the parietal operculum overlapped with the result of functional connectivity analysis, i.e., there was a significant association between the KOOS pain score changes and the corresponding rFPN connectivity to the left operculum/S2 changes after longitudinal treatment (r = 0.48, p = 0.007) (Fig. 3 shown in blue).

To further explore the brain activity changes evoked by acupuncture needle stimulation, we lowered the threshold to P < 0.005 voxel wise uncorrected with 50 voxels. Our results showed that verum acupuncture needle stimulation also produced significant fMRI signal increase in the left insula and putamen compared to sham acupuncture. Using the same threshold, we also found a significant fMRI signal increase in the bilateral operculum/S2 in both high and low dose groups when we compared brain activity during needle stimulation with brain activity at baseline (in the absence of needle stimulation) (Additional file 3: Figure S3). This finding suggests that there was a similar brain activation pattern in the two verum acupuncture treatment groups.

## Discussion

In this study, we investigated the effects of longitudinal acupuncture treatment on brain functional connectivity and clinical outcome measurements in knee OA patients. The results showed that acupuncture treatment can significantly modulate brain functional connectivity and pain as assessed by a KOOS pain subscale in OA patients.

The results from previous studies suggest that acupuncture treatment can significantly modulate FC in healthy subjects [28–30, 32, 33]. However, whether acupuncture treatment can modulate the FC in patient populations, which are often characterized by abnormal brain network functioning and requiring longitudinal treatment, remains to be answered. The results from more recent studies suggest that FC changes after medical treatments are significantly associated with clinical outcome changes [49–51], implying that the investigation of FC changes can be a useful tool in clinical research. In our study, we found that more FC changes between the rFPN and the left insula correlated with less pain after longitudinal treatment. The verum acupuncture group exhibited a stronger increase in connectivity than did the sham acupuncture group after longitudinal treatment.

Previous studies suggest that the insula/operculum/S2 is involved in the perception of pain [52–55] and the frontoparietal network is involved in cognitive control and pain modulation [47, 56]. Downar et al. suggested that the temporal/parietal junction, inferior frontal gyrus, insula and the ACC make up a multimodal cortical network that detects and controls sensory input from the environment [57]. Later, Vincent et al. identified the lateral prefrontal cortex, ACC, insula, and inferior parietal lobule as a frontoparietal control system engaged in cognitive control and decision-making processes [58]. The right lateral frontal cortex, which is part of the rFPN, has exhibited the ability to inhibit pain sensation and modulate pain in previous imaging studies [59, 60].

Anatomically, the posterior parietal cortex, which is also part of the rFPN, is interconnected with the insula, ACC, orbital prefrontal cortex, and parahippocampal cortex and receives projections from the somatosensory cortices [61]. Studies have shown that in knee OA patients, the lateral prefrontal cortex, superior and inferior parietal lobule, rACC, insula, parietal operculum, and limbic cortical areas are involved in altered pain processing [62, 63]. This suggests that the rFPN, rACC, insula, and parietal operculum are the main areas involved in the OA pain circuit. We now show that verum acupuncture modulates activity in these regions more compared to sham.

The ECN located in the anterior of the frontal cortex (including dorsal and medial prefrontal cortex, and anterior cingulate cortex) is involved in integrating information from the external environment with stored internal representations [64], controlling top-down attention during conflict processing of alternative responses [65], and monitoring conflict with subsequent adjustment in performance [66–69].

Following longitudinal treatment, both the rFPN and the ECN showed stronger connectivity with the rACC/MPFC in the verum group compared to the sham group. The rACC/MPFC are key regions involved in the descending pain modulatory system [47, 70–73]. Studies also showed that the rACC is involved in self-regulation of pain such as placebo analgesia [54, 60, 74]. It also forms a core network with the PAG and the RVM (ACC-PAG-RVM network) for pain modulation even in the absence of a painful stimulus [75].

In a previous study, investigators found that the functional connectivity fluctuations and structural connectivity between the PAG and the rACC/MPFC predicted the mind wandering away from pain [76]. In another study [47], we found that pre-test resting state FC between the rFPN and rACC/MPFC can significantly predict the conditioning placebo cue effect, implying that the intrinsic linkage between the two regions is crucial for self-modulation of pain. Similarly, activity in the rACC/MPFC, insula, and PAG was implicated in pain controllability [77]. In knee OA patients, the rACC co-varies with clinical knee OA pain [62], which suggests that the region is the essential node in pain modulation for knee OA patients.

In a more recent study using the same dataset [78], we found that baseline KOOS pain and sport scores were associated with the PAG FC with MPFC and hippocampus. Specifically, lower connectivity between the PAG and MPFC was associated with worse sport function (lower KOOS sport score), while higher PAG/hippocampus connectivity was associated with worse pain (lower KOOS pain score). Verum acupuncture-induced improvement in pain and sport scores (compared to sham) was associated with the modulation of PAG-MPFC and PAG-hippocampus connectivity—increase and decrease, respectively, suggesting the role of PAG in acupuncture-related improvement. This result is also consistent with a another study [79] using the same data set, in which we found acupuncture can prevent the thinning of cortical thickness at the posterior MPFC (a region in ECN) in knee OA patients, and the resting state FC between the posterior MPFC and the key descending pain modulatory system (rACC and PAG) is significantly stronger in real acupuncture as compared with sham after longitudinal treatment.

In light of the above findings and previous studies on the mechanisms involved in acupuncture analgesia [19–21] demonstrating the involvement of the pain descending modulatory system, we speculate that the enhanced connectivity between the rFPN and ECN networks and the rACC/MPFC may suggest that verum acupuncture decreases patients’ pain experience by enhancing the descending pain modulatory system.

In the present study, we found that following longitudinal treatment, FC between the sensorimotor network and the dorsal ACC exhibited a greater decline in the verum group than in the sham group. It is well known that the dorsal ACC is associated with the affective component of pain [55, 60, 80–82] and can be modulated by acupuncture needle stimulation [27]. We speculate that the decrease in the connectivity between these two regions may suggest that acupuncture can modulate (relieve) affective pain experience [83, 84].

In recent years, many investigators have explored acupuncture needle stimulation-evoked brain activity changes using fMRI [23, 24]. A wide range of brain networks are involved in acupuncture stimulation and each region may respond differently to needle stimulation [85]. In this study, we found that during acupuncture needle stimulation, there is stronger activation in the left parietal operculum in verum acupuncture as compared to sham acupuncture, which is consistent with findings from previous studies [23, 24, 26, 86]. A study by Pariente and colleagues [87] compared verum, sham (Streitberger needle) and open placebo acupuncture and found that verum acupuncture activated the insula, DLPFC, rACC, and midbrain compared to open sham; however, only the insula was more activated by verum acupuncture when accounting for expectation (comparison with the Streiberger needle stimulation). Our results are consistent with above findings.

Additionally, we found a positive association in the functional connectivity changes between the rFPN and the left parietal operculum (post–pre) and the corresponding changes in clinical KOOS pain score. These results suggest that lower levels of pain correlate with stronger connectivity between the rFPN and the left posterior parietal operculum after longitudinal acupuncture treatment. Investigators have identified the parietal operculum as one of the main regions related to OA pain [63] and pain perception [88]. Our results suggest that the parietal operculum may be a key region in acupuncture treatment that serves to enhance connectivity between the rFPN and the insula and rACC/MPFC, resulting in increased communication between these regions and restoration of the descending modulatory pathway.

**Fig. 4:**
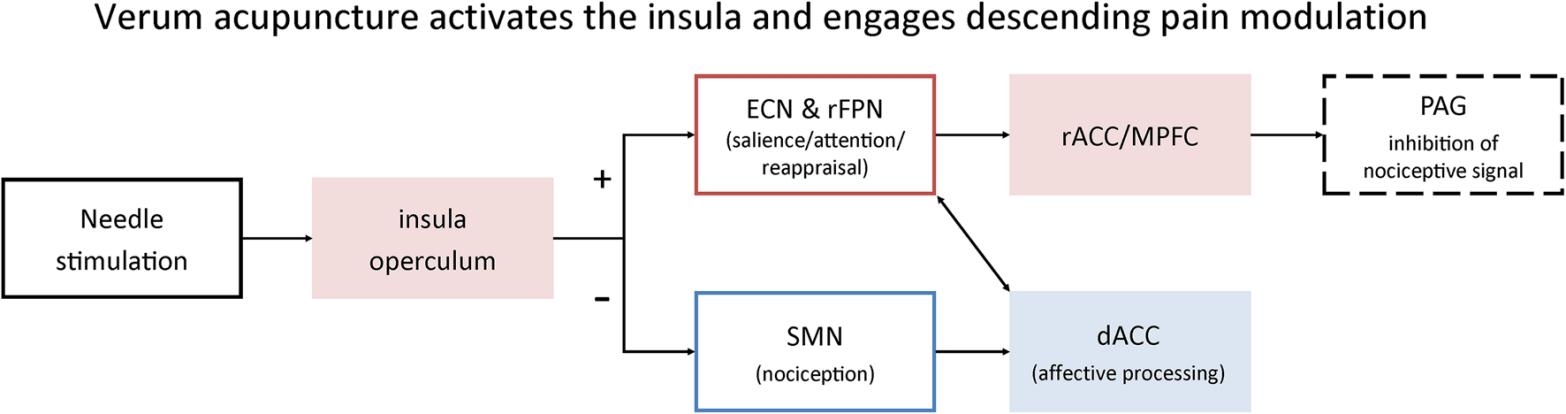
Hypothetical schematic illustration of the resting state functional connectivity modulated by acupuncture and its relevance to pain relief. Note that this diagram does not exhaustively describe all networks and brain regions involved in acupuncture analgesia but only summarizes the mechanisms likely relevant for the current study. *Red* indicates increased activation/connectivity; *blue* indicates decreased activation/connectivity. Verum acupuncture needle stimulation activated the operculum/insula, as suggested by the functional MRI analysis. The insula/operculum processes information about both the sensory component of pain (posterior insula), as well as cognitive-emotional aspects of pain (anterior insula) [101]. In its capacity as a nociceptive salience detection, affective and pain decision-making hub [102], it might (1) increase functional connectivity between the attention, cognitive control and appraisal networks (rFPN, ECN) and the rACC/MPFC, which is a key brain region for attention and descending pain modulation with a direct connection to the PAG [75], further inhibiting noxious input; (2) decrease functional connectivity between the SMN and dACC, representing reduced interaction between the sensory and affective components of pain processing, providing further relief from pain

Summing up, it seems that the insula/operculum activity appeared in all analyses: (1) fMRI signal increase during needling stimulation (verum vs sham); (2) resting state FC group differences, and (3) association between the resting state FC changes and the corresponding KOOS pain changes in all subjects. A review of the insula function [89] suggested that it represents a hub for (1) bottom-up detection of salient stimuli, (2) switching between large-scale attention and memory networks, (3) interacting with the anterior and posterior insula to modulate autonomic reactivity, and 4) coupling with the anterior cingulate to facilitate rapid access to the motor system. We observed brain activity and connectivity patterns consistent with all of these functions during verum acupuncture compared to sham. Therefore, in line with this model, we speculate that verum acupuncture needle stimulation activates the operculum/insula, which triggers the rFPN and ECN. The two networks further activate the descending pain modulatory network through the rACC/MPFC to achieve the modulation effect in the descending pain network (please note that although no differences in the PAG were directly observed in the current study, the intrinsic connectivity of the PAG and rACC/MPFC has been demonstrated in our previous study [75]). In parallel, the coupling between the insula/operculum and dACC modulates the sensory-motor network, interfering with the sensory motor changes evoked by knee pain to relieve pain experience (Fig. 4). Future studies are needed to elucidate the details of the modulation process.

One attribute of this study is that the needles were repeatedly manipulated. This differs significantly from the previous clinical trials [13, 14], which found no significant difference between real and sham acupuncture (in these trials, the needle stimulation is almost not applied). Based on Traditional Chinese Acupuncture theory, deqi (the sensations evoked by acupuncture needle stimulation) is key for the clinical achievement of acupuncture treatment [46]; previous studies [90] also suggested that strong or moderate stimulation is crucial for acupuncture analgesia. This may explain the group differences in KOOS pain observed with a relatively small sample size. Nevertheless, we would like to emphasize that our result is very preliminary and further study with a larger sample size is needed to validate this finding.

In this study, we also attempted to explore the effect of acupoint number. With a small sample size, our preliminary result suggested that if the total stimulation amount remains the same, the treatment effect and brain activation pattern is also similar in knee OA patients. Further study is needed to validate the findings with large sample size in different patient populations.

Several limitations of this study should be mentioned. First, the sample size is relatively small. However, we made several steps to increase the power in the following ways. On the one hand, by combining the two acupuncture groups, we increased the power of the verum group, creating a 2:1 ratio between the verum and sham treatment conditions that provided additional information on patients receiving active treatment [91]. On the other hand, due to the longitudinal design of the study, we acquired fMRI scans at three time points, which may have also increased the power of this study. All reported main brain imaging results survived the stringent multiple comparison correction.

Another limitation of this study was the potentially confounding effect of factors such as patient age and duration of chronic pain experienced by each patient. To address this concern, we repeated the analysis including age and duration of pain as covariates of no interest and re-analyzed the data. After this analysis, most of the key findings remained significant. Thus, our main results do not change after adjusting for these confounding factors. Furthermore, when KOOS pain scores were correlated with age and duration using multiple regression, we did not find any significant correlation. Further studies with larger sample sizes are needed to validate the findings of the study.

Thirdly, patients with knee OA very commonly report pain in other regions, including the neck and low back, and the contralateral knee and hips, which may also have influenced brain FC. However, we can assume these conditions were controlled for after randomization. In addition, the comparisons in this study were applied between pre- and post-treatment differences. Thus, we expect that these potential confounding factors should not influence the conclusions of this study.

Fourth, there are two patients who dropped in each real acupuncture treatment group. Due to a small sample size, this could potentially have influenced the conclusion of our study. Nevertheless, as we pointed out above, of the four patients who withdrew, one subject was unable to adhere to study requirements in the scanner and three had scheduling conflicts (the fact that the fMRI treatment session can only take place in certain time intervals significantly limited the flexibility of this study). None of these reasons, to the best of our knowledge, was related to the clinical outcome of treatments. We therefore believe it did not influence the validity of the study.

Finally, all subjects agreed not to use their regular medication during the acupuncture treatment period, except for painkillers as needed. However, we cannot completely exclude the potential influence of other treatments.

## Conclusion

Our findings suggest that verum acupuncture may enhance connectivity in the descending pain modulation pathway through several networks, including the rFPN and ECN. A better understanding of the association between FC and clinical outcomes and how treatment can modulate FC may ultimately lead to the acceptance of acupuncture in mainstream medicine, and facilitate the development of mechanism-based therapies for chronic pain.

## Methods

We briefly describe the experimental procedures below. Please also see previously published studies [45, 78, 79] for more details on the experimental procedure. The data has been used in previous studies to investigate the reliability of deqi sensation evoked by acupuncture needle stimulation [45], cortical thickness changes after longitudinal real and sham acupuncture treatments [79], and resting state FC changes of the PAG [78]. In this study, we used ICA to investigate the resting state FC changes before and after longitudinal acupuncture treatments, as well as the brain response during acupuncture needle stimulation. These results have not been reported before.

### Subjects

The Institutional Review Board at Massachusetts General Hospital approved all study procedures. Subjects who enrolled in this study provided written informed consent before beginning any study procedures. We debriefed all subjects at the end of the study.

### Patient recruitment and inclusion criteria

We recruited acupuncture naïve patients aged 40–70 with a diagnosis of chronic osteoarthritis (OA) in the right and/or left knee from Massachusetts General Hospital (MGH) and Brigham and Women’s Hospital (BWH).

Subjects exhibited Grade 2 or Grade 3 OA as defined by the Kellgren-Lawrence Scale used for radiographically grading knee OA. We excluded subjects who: (1) had undergone any interventional procedures for knee pain within 6 months prior to enrolling in the study, (2) intended to undergo surgery during the time of involvement in the study, (3) experienced knee pain due to causes other than OA, such as inflammation or malignancy, (4) had received a diagnosis of rheumatoid arthritis or other leg-related pain disorders, (5) were taking opioids, benzodiazepines, or other medications that may influence brain imaging outcome, or (6) presented MRI contraindications. All enrolled OA patients had an endogenous average pain intensity rating of >2 on the Brief Pain Inventory (BPI) scale at the first visit.

### Experimental design

All subjects, regardless of the presence of bilateral or unilateral pain, received treatment on the knee that presented them with the most severe pain. We stratified subjects by knee and randomized them into one of three groups: high dose real acupuncture (6 acupoints), low dose real acupuncture (2 acupoints), or sham acupuncture (6 non-acupoints with Streitberger placebo needles [92]) (Additional file 4: Figure S4). We used the permuted block randomization stratified by the acupuncture-treated knee. The randomization number was provided by a biostatistician and only the acupuncturist was not blinded to the study randomization before the end of the study. The randomization was applied after the QST measurement and psychometric assessment in session 1. This time was chosen because the different acupuncture exposure took place immediately afterwards.

In this study, we were interested in whether the number of needles (acu points) can influence the treatment effect. Therefore, we had two groups of real acupuncture treatments with different numbers of acupoints, but an identical stimulation paradigm.

After an initial screening session, each subject engaged in a total of 6 acupuncture treatment sessions in 1 month (twice per week for the first 2 weeks, once per week for the last 2 weeks). Treatments 1, 3 and 6 occurred during a scan session with the patient lying in a 3-Tesla MRI scanner while we acquired fMRI data. All other treatments occurred in our behavioral testing room.

### Acupuncture administration

Each acupuncture treatment session for subjects in both the verum and sham acupuncture groups lasted about 25 min. The two acupoints selected for the low dose acupuncture group [ST35 and Xi yian (extra point)] were located near the knee and each acupoint was stimulated a total of twelve times. The high dose acupuncture group received treatment at four additional acupoints (GB34, SP9, GB39 and SP6) (see Additional file 1: Figure S1b) and each acupoint was stimulated 4 times. All these points are well documented for the treatment of knee pain [8, 10, 11].

For subjects receiving sham acupuncture, the acupuncturist applied placebo needles [54, 92–94] at six non-acupoints using a paradigm identical to that of the high dose verum needle administration. Rather than penetrating the skin like a verum needle, the sham needle retracts up the handle shaft when the acupuncturist presses it against the skin. Sham point 1 was located 1.5 cun posterior and inferior to GB4, sham points 2–3 were located 1.5 and 3 cun inferior to sham point 1, sham point 4 was located 1 cun posterior to the midpoint of K9 and K10, and sham 5–6 were located 1.5 cun inferior and superior to the sham point 4, respectively. All sham points were located on the lower leg where no meridians pass (Additional file 1: Figure S1b).

For all treatments, the acupuncturist placed a small plastic ring over the point and secured the ring with a thin strip of sterile plastic tape. This ensured patient blindness to the actual site of needle insertion and thus blindness to whether the treatment was verum or sham. During acupuncture, the acupuncturist stimulated one point at a time in a predetermined order, each for 10 s with 30-s breaks between each acupoint. (Additional file 1: Figure S1a). The total treatment and stimulation time for all three groups was the same. We randomized the specific starting acupoint across patients, but held it constant throughout all sessions for each individual patient. For consistency, we kept leg position, acupoint location, and needling parameters (1–2 cm depth, approximately 120 rotations/min, and moderate deqi sensations on a 0–10 scale) constant across groups. In fMRI session, there were two acupuncture scans per treatment, with each lasted for 9 min, followed by Massachusetts General Hospital Acupuncture Sensation Scale (MASS) rating.

As a treatment quality control, we measured the deqi sensation evoked by two separate acupuncture treatments (one midway through the treatment and one at the end of the treatment) using the MASS [45, 46].

### Clinical outcomes

#### Knee injury and Osteoarthritis Outcome Score (KOOS)

We measured clinical outcomes using the KOOS [44], which is comprised of 5 subscales: (1) pain, (2) other symptoms, (3) function in daily living (ADL), (4) function in sport and recreation, and (5) knee-related quality of life (QOL). Each subscale allows for calculation of a normalized score, with 0 denoting the most extreme symptoms/pain and 100 denoting no symptoms/pain [44]. Based on previous studies [8], we selected the KOOS pain subscale and function in daily living subscale as our primary outcome measures. We used all other subscale measurements as secondary outcomes. Trained research assistants, blinded to treatment mode, administered the KOOS to all patients at baseline and again after the final treatment.

### fMRI data acquisition

Each subject participated in three identical fMRI scanning sessions. Scan 1 (treatment 1) and scan 2 (treatment 3) were separated by approximately 7 days; scan 2 and scan 3 (treatment 6) were separated by approximately 14 days. We used a 3-axis gradient head coil in a 3-Tesla Siemens MRI system equipped for echo planar imaging (EPI). Each fMRI session included an anisotropic magnetization prepared rapid gradient-echo (MPRAGE) structural sequence followed by a 6-min resting state fMRI. The MPRAGE scanning parameters included TR of 2200 ms, echo time of 9.8 ms, flip angle of 7, field-of-view of 230 mm^2^, slice thickness of 1.2 mm. For the resting state, the scan acquisition included 47 slices with a thickness of 3 mm, a TR of 3000 ms, a TE of 30 ms, flip angle of 85°, field of view of 216 mm^2^ and a 3 × 3-mm in-plane spatial resolution. Following the first resting state fMRI, we conducted two functional scans during the 25-min acupuncture administration and a second resting state scan following acupuncture administration (Additional file 1: Figure S1a).

### Data analysis

#### Independent component analysis for resting state fMRI data

We analyzed pre- and post-acupuncture resting state data for treatments 1, 3 and 6 using independent component analysis in FMRIB Software Library (FSL) [95], following the same processing steps as described in a previous study [47, 48]. We first applied a band pass filter between 0.01 and 0.1 Hz to the functional time series corrected for motion using MCFLIRT and slice timing, skull stripped using the Brain Extraction Tool (BET), and smoothed (full width at half maximum = 5 mm). Then we registered the data to their respective skull stripped anatomical volume and further registered to the MNI152 template using linear affine transformations with 12° of freedom. We then concatenated the functional data into 4D data and performed a probabilistic independent component analysis using Multivariate Exploratory Linear Optimized Decomposition into Independent Components (MELODIC) [96] on the data set to identify 20 resting state networks. We used an algorithm to search for similarities between our group-level networks and the template networks derived from 1414 healthy subjects to identify the corresponding network for our results [48].

We chose the sensory-motor network (SMN), executive control network (ECN), and right frontoparietal network (rFPN) for further analyses. Previous ICA studies have reported that these networks have been reliably observed and are believed to be associated with pain process and modulation [47, 56] as well as development of chronic pain [97, 98].

To perform group-level analyses of the association between subjects’ responses to acupuncture and resting state networks derived from ICA, we used a dual-regression technique [48]. In brief, we used the three previously defined networks as spatial regressors in a general linear model (GLM) to extract temporal dynamics associated with each spatial map. The resulting time courses served as temporal regressors in a GLM to generate subject-specific-maps of the whole brain for each subject.

Finally, group analyses were performed using the whole-brain subject-specific network maps from the second GLM. The results represent the strength of FC for each voxel within each of the chosen networks.

To explore the association between the clinical outcomes and FC changes, we performed regression analyses using the network connectivity “change maps” between pre-acupuncture treatment 1 and pre-acupuncture treatment 6 resting state scans and change in clinical outcome (KOOS pain score post–pre). To remove any baseline effect, we input all changes in KOOS pain scores as percent change [(*post*–*pre/pre*) × 100 %].

To investigate the modulation effect of acupuncture treatment, we compared the changes in pre-acupuncture treatment 6 and pre-acupuncture treatment 1 in the verum group to that of the sham group. We used pre-acupuncture resting state fMRI to avoid the residual effect of acupuncture needle stimulation [99, 100]. Due to the small sample size of each group (10 patients), we decided to combine the two verum acupuncture groups (low and high dose groups) because (1) the total treatment time, needle stimulation time, and reported sensations were the same in both verum groups; (2) we found no clinical outcome differences between the two verum acupuncture groups and both verum acupuncture groups showed a trend towards improvement when compared to sham treatment, and (3) high and low dose acupuncture needle stimulations evoked similar activation patterns (Additional file 3: Figure S3)**; (**4) reported sensations as measured by MASS were similar in both real acupuncture treatment groups, and both showed significant differences from the sham acupuncture group; (5) acupuncture prescription for Knee OA varies across different studies; combining two protocols may better represent clinical acupuncture in practice; and (6) combining two groups gave us more power to test our hypothesis. We performed all resting state analyses with a voxel-wise cluster forming threshold of Z > 2.3 and a corrected cluster significance threshold of P < 0.05.

To test the effect of acupuncture treatment across all three sessions, we extracted the peak Z values of each significant network from previous steps for both verum and sham group and performed repeated measure ANOVA with each treatment session as one time point using SPSS 18.0 Software (SPSS Inc., Chicago, IL, USA).

To control for the potentially confounding factors including age and duration of knee OA, we also repeated the above analysis including age and duration of pain as covariates of no interest.

#### Task-related fMRI data analysis

To explore how acupuncture needle stimulation affected brain activity changes, we performed data analysis using Statistical Parametric Mapping (SPM8, Wellcome Trust Centre for Neuroimaging, London, UK). During the preprocessing, we realigned all functional volumes, spatially normalized, and smoothed using an 8-mm full-width at half-maximum Gaussian kernel.

We used a general Linear Model (GLM) for first level analysis. Each individual design matrix contained the regressor for the main effect of acupuncture, combining acupuncture sessions 1 and 2 for each treatment session. To evaluate the effect of verum greater than sham acupuncture, we performed second level analyses using a full factorial design with two factors. The first factor had two levels (high and low dose verum acupuncture, and sham acupuncture) and the second factor had three levels (treatment 1, treatment 3 and treatment 6). By pooling all 3 treatment sessions together for each group, we estimated the effect of verum acupuncture as compared with sham acupuncture in the second level analyses. To further explore the brain activation evoked by needle stimulation during high dose and low dose acupuncture, we also calculated the fMRI signal change for each group compared to baseline separately. A threshold of voxel wise p < 0.001 uncorrected and p < 0.05 corrected (family-wise error, FWE) at cluster level was used for data analysis.

## Additional files

10.1186/s12990-015-0071-9 Acupuncture Protocol **A)** A 25-min acupuncture treatment scan was acquired between two 6-min resting state scans. The acupuncture stimulation paradigm (verum and sham) indicates the timeline of needle stimulation during treatment. (M = manual acupuncture) The total treatment duration and stimulation time is identical between all three groups. **B)** Verum and sham acupuncture points. Low dose acupoints: ST35 and Xi yian; high dose acupoints include low dose acupoints and GB34, SP9, GB39 and SP6.
10.1186/s12990-015-0071-9 ICA identified 20 resting networks. The three networks used in this study include the right frontoparietal network, the executive control network, and the sensory-motor network.
10.1186/s12990-015-0071-9 Similar positive brain activations regions associated with acupuncture needle stimulation in high and low dose acupuncture treatment groups. A threshold of p < 0.005 with 50 continuous voxels was applied for visualization. A) High dose: left parietal operculum with peak of −66, −31, 40 (x, y, z), right parietal operculum (63, −28, 25) B) Low dose: left parietal operculum (−56, −28, 25), right parietal operculum (60, −24, 25).
10.1186/s12990-015-0071-9 Study design. (44 subjects recruited, 30 subjects were scanned). Subjects randomized into high dose, low dose, or placebo group (N = 10 for each group).
