# Supplementary figures and images for: The modulation effect of longitudinal acupuncture on resting state functional connectivity in knee osteoarthritis patients
Source: Mol Pain. 2015 Oct 29;11:67. doi: 10.1186/s12990-015-0071-9 (PMC4625557; doi:10.1186/s12990-015-0071-9)

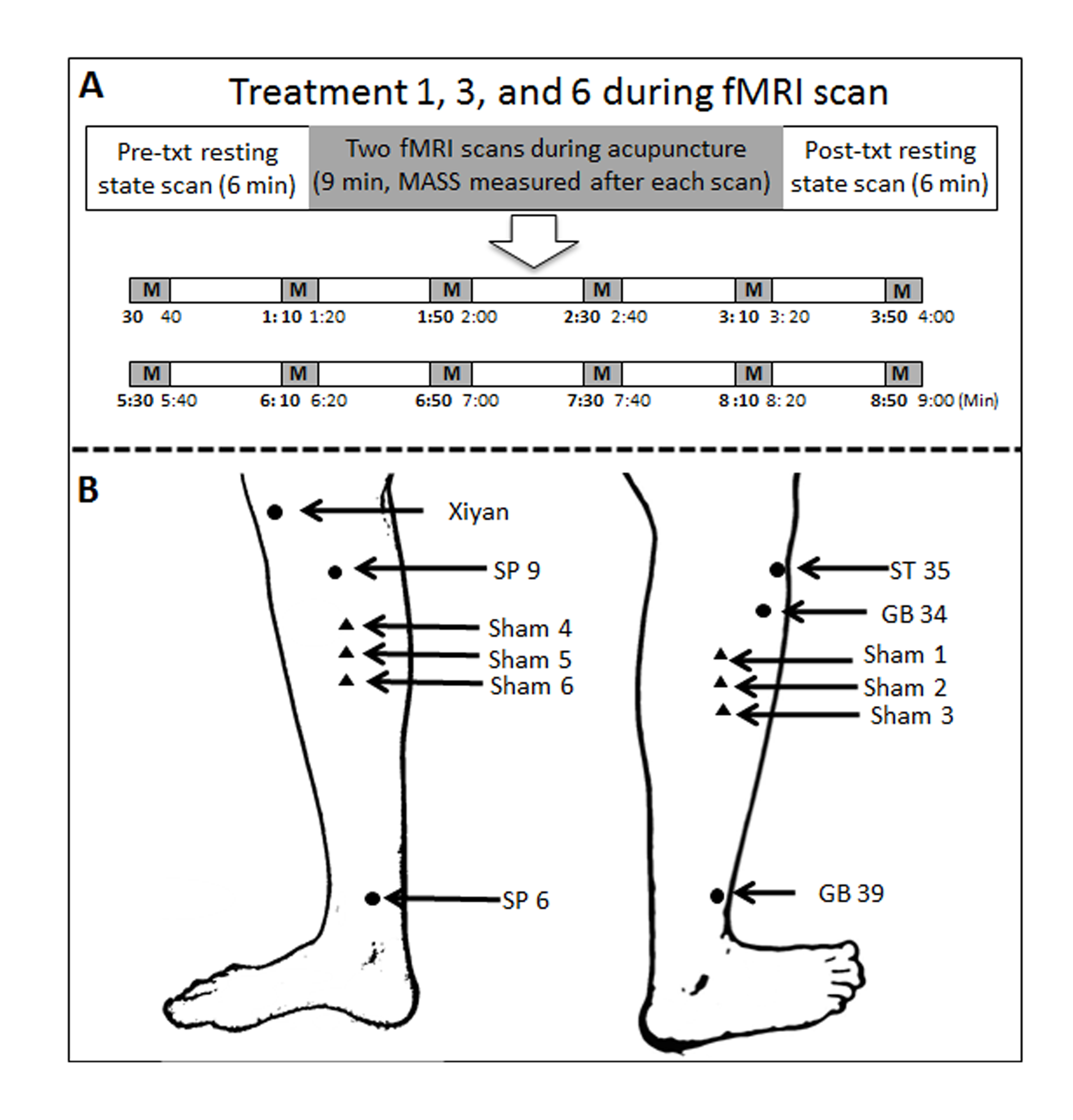

Supplement: Supplementary file 1 — 10.1186/s12990-015-0071-9 Acupuncture Protocol A) A 25-min acupuncture treatment scan was acquired between two 6-min resting state scans. The acupuncture stimulation paradigm (verum and sham) indicates the timeline of needle stimulation during treatment. (M = manual acupuncture) The total treatment duration and stimulation time is identical between all three groups. B) Verum and sham acupuncture points. Low dose acupoints: ST35 and Xi yian; high dose acupoints include low dose acupoints and GB34, SP9, GB39 and SP6. [file 12990_2015_71_MOESM1_ESM.jpg]

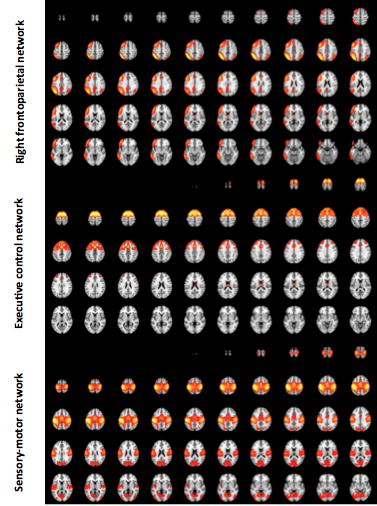

Supplement: Supplementary file 2 — 10.1186/s12990-015-0071-9 ICA identified 20 resting networks. The three networks used in this study include the right frontoparietal network, the executive control network, and the sensory-motor network. [file 12990_2015_71_MOESM2_ESM.tiff]

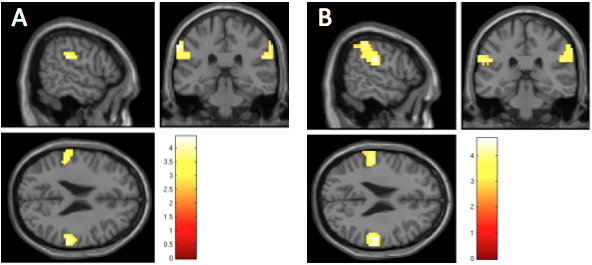

Supplement: Supplementary file 3 — 10.1186/s12990-015-0071-9 Similar positive brain activations regions associated with acupuncture needle stimulation in high and low dose acupuncture treatment groups. A threshold of p < 0.005 with 50 continuous voxels was applied for visualization. A) High dose: left parietal operculum with peak of −66, −31, 40 (x, y, z), right parietal operculum (63, −28, 25) B) Low dose: left parietal operculum (−56, −28, 25), right parietal operculum (60, −24, 25). [file 12990_2015_71_MOESM3_ESM.tiff]

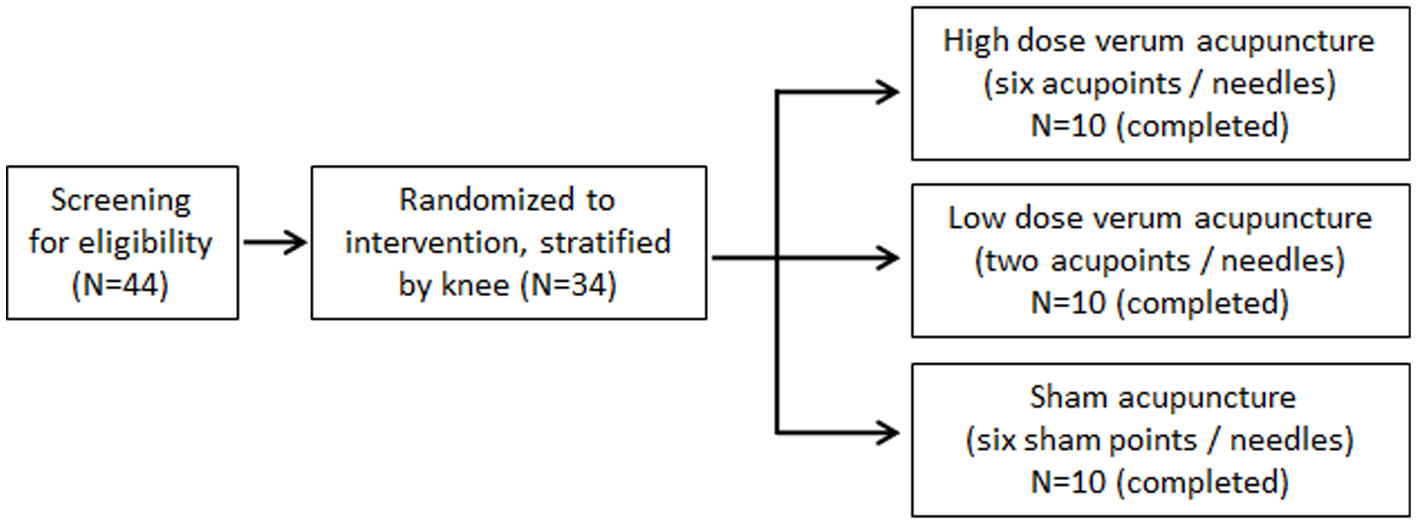

Supplement: Supplementary file 4 — 10.1186/s12990-015-0071-9 Study design. (44 subjects recruited, 30 subjects were scanned). Subjects randomized into high dose, low dose, or placebo group (N = 10 for each group). [file 12990_2015_71_MOESM4_ESM.jpg]
